# Supplementary figures and images for: Activated Alpha 2-Macroglobulin Is a Novel Mediator of Mesangial Cell Profibrotic Signaling in Diabetic Kidney Disease
Source: Biomedicines. 2021 Aug 30;9(9):1112. doi: 10.3390/biomedicines9091112 (PMC8471248; doi:10.3390/biomedicines9091112)

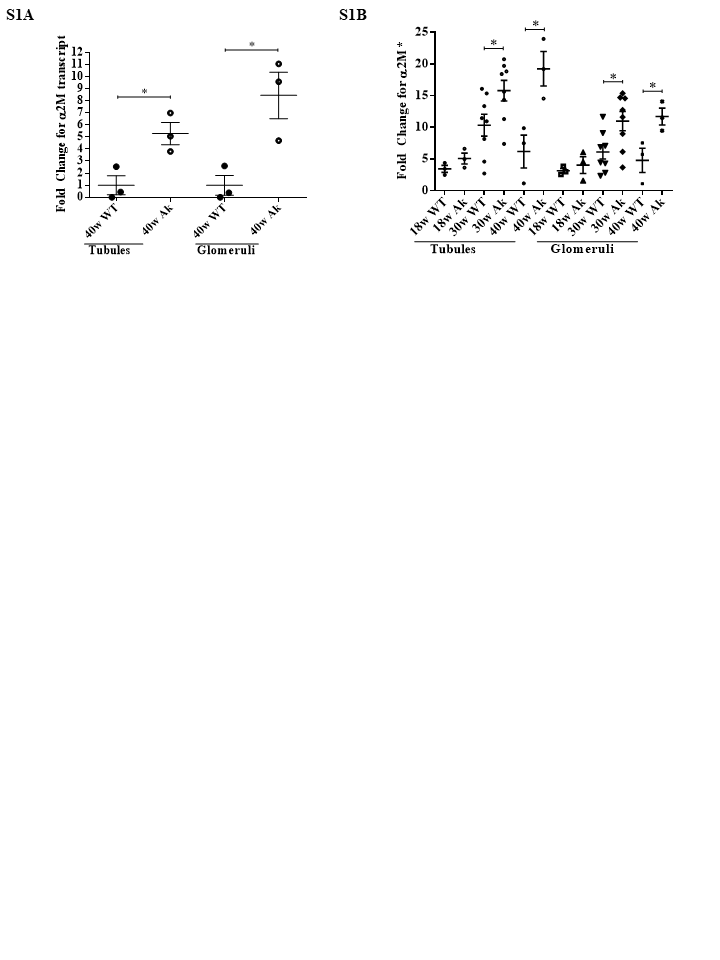

Supplement: Supplementary file 1 [file biomedicines-09-01112-s001.zip › Supplementary Figures/Figure S1.TIF]

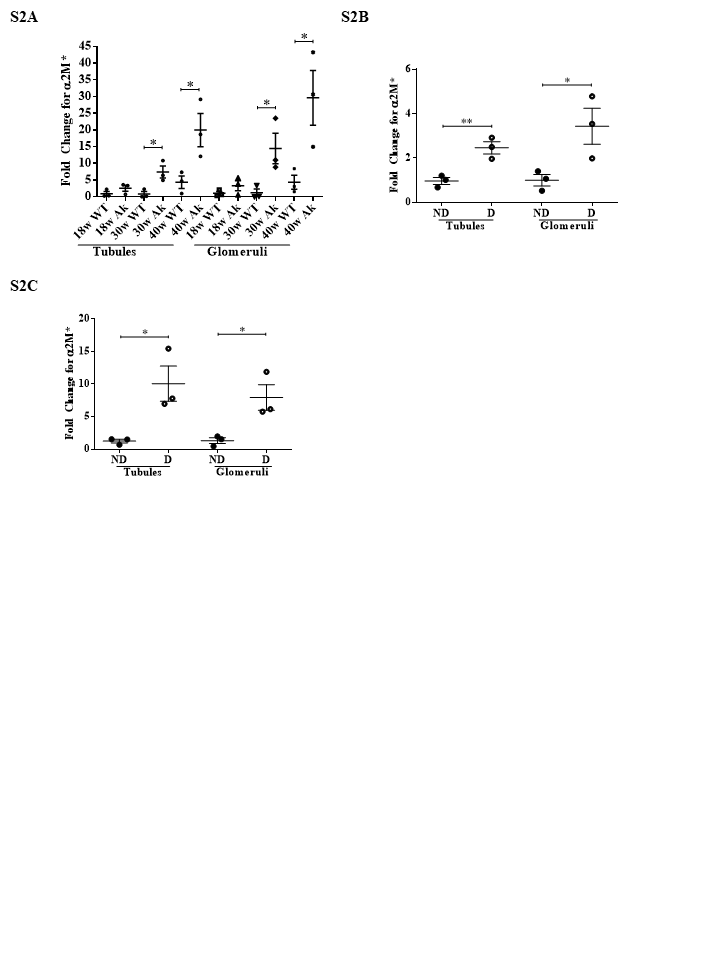

Supplement: Supplementary file 1 [file biomedicines-09-01112-s001.zip › Supplementary Figures/Figure S2.TIF]

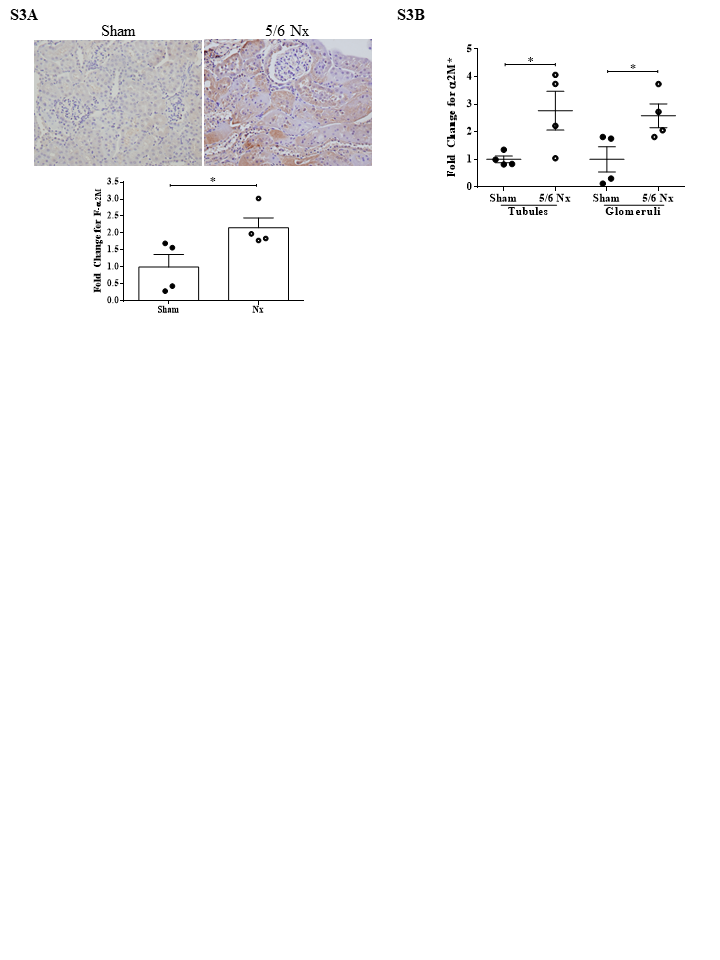

Supplement: Supplementary file 1 [file biomedicines-09-01112-s001.zip › Supplementary Figures/Figure S3.TIF]
